# Transitions of care for older adults discharged home from the emergency department: an inductive thematic content analysis of patient comments

**DOI:** 10.1186/s12877-023-04482-0

**Published:** 2024-01-03

**Authors:** Vanessa Couture, Nathalie Germain, Émilie Côté, Lise Lavoie, Joanie Robitaille, Michèle Morin, Josée Chouinard, Yves Couturier, France Légaré, Marie-Soleil Hardy, Lucas B. Chartier, Audrey-Anne Brousseau, Nadia Sourial, Éric Mercier, Clémence Dallaire, Richard Fleet, Annie Leblanc, Don Melady, Denis Roy, Samir Sinha, Marie-Josée Sirois, Holly O. Witteman, Marcel Émond, Josée Rivard, Isabelle Pelletier, Stéphane Turcotte, Rawane Samb, Raphaëlle Giguère, Lyna Abrougui, Pascal Y. Smith, Patrick M. Archambault, on behalf of the Network of Canadian Emergency Researchers

**Affiliations:** 1 Centre de recherche intégrée pour un système apprenant en santé et services sociaux, Centre intégré de santé et services sociaux de Chaudière-Appalaches, Lévis, Québec Canada; 2 Faculty of Medicine, Université Laval, Québec, Québec Canada; 3 Department of Social Work, Université de Sherbrooke, Sherbrooke, Québec Canada; 4 VITAM - Centre de recherche en santé durable, Québec, Québec Canada; 5 Department of Family Medicine and Emergency Medicine, Université Laval, Québec, Québec Canada; 6 Centre de recherche du CHU de Québec - Université Laval, Axe santé des populations et pratiques optimales en santé, Université Laval, Québec, Québec Canada; 7 Department of Emergency Medicine, University Health Network, Toronto, ON Canada; 8 Department of Family Medicine, Université de Sherbrooke, Sherbrooke, Québec Canada; 9 Department of Health Management, Evaluation and Policy, School of Public Health, Université de Montréal, Montréal, Québec Canada; 10 Faculty of Nursing Science, Université Laval, Québec, Québec Canada; 11 Department of Family and Community Medicine, University of Toronto, Ontario, Canada; 12 Schwartz-Reisman Emergency Medicine Institute, Mount Sinai Hospital, Toronto, ON Canada; 13 Commissaire à la santé et au bien-être (CSBE), Québec, Québec Canada; 14 Department of Medicine, University of Toronto, Toronto, Canada; 15 Department of Medicine, Sinai Health System and University Health Network, Toronto, ON Canada; 16 Département de réadaptation, Faculté de médecine, Université Laval, Québec, Québec Canada; 17 Faculty of Science and Engineering, Université Laval, Québec, Québec Canada; 18 Network of Canadian Emergency Researchers, Ottawa, ON Canada

**Keywords:** Emergency department, Care transitions, Patient experience, Aging, COVID-19, Geriatrics

## Abstract

**Objective:**

Improving care transitions for older adults can reduce emergency department (ED) visits, adverse events, and empower community autonomy. We conducted an inductive qualitative content analysis to identify themes emerging from comments to better understand ED care transitions.

**Methods:**

The LEARNING WISDOM prospective longitudinal observational cohort includes older adults (≥ 65 years) who experienced a care transition after an ED visit from both before and during COVID-19. Their comments on this transition were collected via phone interview and transcribed. We conducted an inductive qualitative content analysis with randomly selected comments until saturation. Themes that arose from comments were coded and organized into frequencies and proportions. We followed the Standards for Reporting Qualitative Research (SRQR).

**Results:**

Comments from 690 patients (339 pre-COVID, 351 during COVID) composed of 351 women (50.9%) and 339 men (49.1%) were analyzed. Patients were satisfied with acute emergency care, and the proportion of patients with positive acute care experiences increased with the COVID-19 pandemic. Negative patient comments were most often related to communication between health providers across the care continuum and the professionalism of personnel in the ED. Comments concerning home care became more neutral with the COVID-19 pandemic.

**Conclusion:**

Patients were satisfied overall with acute care but reported gaps in professionalism and follow-up communication between providers. Comments may have changed in tone from positive to neutral regarding home care over the COVID-19 pandemic due to service slowdowns. Addressing these concerns may improve the quality of care transitions and provide future pandemic mitigation strategies.

**Supplementary Information:**

The online version contains supplementary material available at 10.1186/s12877-023-04482-0.

## Introduction

Older adults are frequent users of emergency departments (EDs) [1, 2]. Two-thirds of ED visits among older adults result in discharge back to the community [3]. Care transitions from the ED are critical moments where vulnerable older adults are at high risk of discharge-related adverse events, which can result in unplanned readmissions and loss of physical, functional, and/or cognitive capacity [3, 4]. Care transitions include sets of actions, both before and after a hospital or ED stay, put in place to ensure the continuity of care [5, 6]. Care transitions are frequent from the ED and represent an important opportunity for quality improvement [7]. They are also costly, necessitating investment of time and resources from clinicians and informal caregivers [8].

The COVID-19 pandemic has also been devastating for older adults’ care transitions [9]. COVID-19 has disproportionately affected older adults with higher risks of complications and mortality [10]. Fear of contracting the virus and preventative measures to reduce their risk of infection have also led to their social isolation [11]. Care transitions during the pandemic have been complicated by the lack of qualified personnel, care transition nurses and social workers being called upon to manage COVID-19 units, caregivers being asked to stay away from hospitals and caregivers becoming ill themselves [12].

Studies published before the pandemic have documented patients’ views about post-ED discharge outcomes [13], but to our knowledge few studies have documented the impact of the pandemic on patients’ experience about their care transitions to improve care transitions in the post-COVID-19 period [14]. Tailoring care transition interventions to the needs of patients, especially those with multi-morbidity, by paying attention to their lived care experiences can help co-create interventions that are patient-oriented and contribute to better outcomes [15]. While some care transition studies have employed patient-centered outcomes [16–23] none have studied interventions that were patient-oriented. Using comments provided by patients, we may be able to identify paths to developing patient-oriented interventions in older adults to improve care transition interventions. The goal of this study was to better understand patients’ care transition experiences that could be used in designing future patient-oriented interventions to improve care transitions in the post-COVID-19 context.

## Methodology

### Study design and context

Using an inductive approach with no a-priori coding scheme [24, 25], we designed this qualitative descriptive study to analyze data collected directly from older patients having experienced care transition before and during pandemic. This study was nested within the LEARNING WISDOM (Supporting the Creation of a LEARNing INteGrated Health System to Mobilize Context-adapted Knowledge With a Wiki Platform to Improve the Transitions of Frail Seniors From Hospitals and Emergency Departments to the cOMmunity) longitudinal cohort study [26]. We followed the Standards for Reporting Qualitative Research (SRQR) to report our results [27]. The protocol for this study was approved by the *Centre intégré de santé et de services sociaux de Chaudière-Appalaches* (CISSS-CA) Ethics Review Committee (project #2018 − 462, 2018-007). The LEARNING WISDOM cohort included older adults who underwent a transition of care following a visit to one of the four EDs in the CISSS-CA (Québec, Canada) between January 2019 and December 2021. The CISSS-CA is an integrated health organization consisting of four acute care hospitals (Hôtel-Dieu de Lévis (HDL), Hôpital de Montmagny (HDM), Hôpital de Saint-Georges (HSG), Hôpital de Thetford-Mines (HTM)). The HDL is a university-affiliated teaching hospital receiving 70,000 visits to the ED annually. The other three sites each receive 30,000 annual ED visits.

### Selection of participants

The LEARNING WISDOM cohort included patients discharged from one of the four EDs before the COVID-19 pandemic and throughout waves one through five of the pandemic in Québec [28]. Recruitment for the LEARNING WISDOM cohort finished on December 21, 2021. Participants were aged 65 years or older and had been discharged back to the community from the ED observation unit. In the four participating EDs, patients triaged to an observation unit are still considered outpatients being considered for admission to hospital or discharge with primary or specialist care follow-up if needed. Patients triaged to a stretcher in the observation unit are often put on close monitoring. Patients only seen in the ambulatory care section of the ED, admitted to hospital, transferred to another hospital, or transferred to a long-term care center were excluded. Participants having already participated in our study were also excluded. Patients had to be able to understand French and provide informed consent. For the full duration of the study recruitment period, at each participating hospital, a list of eligible discharged patients was generated each day. Patients were called using a computer-generated randomized list for each day of the month and patients were contacted in order until three to five patients were enrolled that day.

### Data collection

As part of a continuous quality improvement project lead by the CISSS-CA participants were contacted by telephone between 24 h and up to seven days after ED discharge, to administer the three-item Care Transitions Measure (CTM-3) [29] (Appendix A), a validated questionnaire that assesses the patient’s opinion about the quality of their care transition [30]. The CTM-3 has three items that investigate three different domains related to the quality of care transitions after discharge from hospital: (1) Whether the patient and family’s preferences were accounted for in the care plan; (2) Whether patients understood their role in self-management after discharge; and (3) Whether patients were briefed on their medications and how to use them before discharge. Patients are asked to qualify their level of agreement with each of the three CTM-3 items (strongly disagree; disagree; agree; strongly agree, missing (don’t know/don’t remember/not applicable)). After completing the CTM-3, patients were then invited to participate in a more in-depth research interview in the following days.

During this second call, patients were required to summarize—in their own words—their understanding of the study to consent, based on the Nova Scotia Criteria [31]. We then asked participants to report on their care transition experience from the ED to home using a structured interview with closed-ended questions and one open-ended response question. We collected sociodemographic information including age, sex, race, language, educational level, family income, pre-consultation living situation, and reason for ED consultation and one single open-ended question asking about their care transition experiences. Patients were asked: *Do you have any details to provide related to your experience of transition from emergency care to returning to your living environment*? Patients answered in as much or as little detail as they wished. We instructed research professionals to collect patients’ comments on all issues concerning their care transitions even if patients commented on their care before arrival to the ED, during their ED stay or after their ED discharge. Our rationale was that elderly patients’ complete care experience during their whole journey to and from the ED, and during their ED stay are important to understand when planning care transition quality improvement interventions.

We refer to these open-ended responses hereafter as comments. A member of the research team asserted the patient’s consent, administered questionnaires, and were instructed to immediately record key elements of each patient’s response to the open-ended question (including important verbatim excerpts) in REDCap (Research Electronic Data Capture [32, 33]). These research professionals were MD students, PhD students in psychology, and research nurses who were trained by the research team coordinator to conduct the interviews and collect important verbatims. This training involved role-playing, simulated calls between the data collection trainees, and direct observation of the first calls until the data collection procedure was respected. Although we did not audio record the calls or produce verbatim transcripts for each call, research professionals were instructed to capture the key open-ended comments made by each patient in short sentences and support these comments with verbatim quotes whenever possible. This data collection process was authorized by the Director of Nursing and the Professional Services Director. Care partners did not provide comments analyzed in this study, nor were they interviewed as proxies to capture patients’ individual care transition experience. The data were anonymized, and a single unique numerical identifier was created for each participant. The CTM-3 in addition to the open-ended question in French and translated in English for this article are presented in Appendix A.

### Quantitative analyses

We used descriptive statistics to present the number of participants who responded to each response category (strongly disagree, disagree, agree, strongly agree, missing (don’t know/don’t remember/not applicable) stratified by pandemic period (pre vs. during COVID-19). To calculate individual CTM-3 scores, we used a linear transformation of its four-point response structure (strongly disagree = 1 point; disagree = 2 points; agree = 3 points; strongly agree = 4 points) to create a CTM-3 score ranging from 0 to 100 [34]. Patients with “missing” results were excluded from analyses and the score was simply calculated by adding the sum of the scores from each item divided by the number of questions answered. We compared mean CTM-3 scores for pre vs. during COVID-19 participants using an independent *t*-test. We also conducted *X* ^*2*^ analyses comparing dichotomous CTM-3 responses for both periods. Values labelled as “missing” were not included in the *X* ^*2*^ analyses.

### Qualitative analyses

Our analysis plan followed an inductive content analysis approach, which consisted of iteratively developing codes used to label the data *during* the process of coding, based on the content of the data set [35, 36]. This approach is well suited to analyzing comments, consisting of interpreting not just the content of the text, but also the interrelations between themes and concepts [35]. This thematic analysis was performed by two female evaluators from different scientific backgrounds (VC, experienced research professional and NG, a Master of Science candidate in epidemiology with training in mixed methods) and supervised by an experienced researcher in qualitative analyses (PMA).

#### Constructing the coding scheme

Patient comments were stripped of any identifying information and placed in a password-protected online collaborative spreadsheet program (Google Sheets, Google LLC, Mountain View, CA) to support thematic analysis. Both evaluators then used a basic inductive content analysis to screen all patient comments in the shared spreadsheet to identify and group overarching keywords into codes in a bottom-up fashion, and then groups of linked codes were clustered into themes. Each theme was reviewed to ensure that it reflected all its associate sub-themes. For instance, a mention of “bed sheets” in patient comments was determined to fall into a subcategory of “bedding, clothing and furniture”, which was under a larger category relating to the patient’s “conditions of stay” at the ED.

These themes and sub-themes were integrated together into a *Mind Map* diagram drawn with *diagrams.net* to guide the coding of individual comments by both evaluators. Throughout the analysis, disagreements and questions between both evaluators were validated by a third author (PMA). Pair debriefing between both evaluators, triangulation (both evaluators coded independently of one another), and expert validation by a third evaluator (PMA) minimized the influence of subjectivity and preconceptions among the coders.

Once the *Mind Map* diagram and coding scheme was developed to map the whole care transition continuum, both evaluators each coded an identical random sample of 40 comments to determine inter-rater reliability. Both reviewers assessed separate online spreadsheets that were then used to independently collect codes (either 1 or 0; each column indicated its own theme) indicating the presence or absence of a theme embedded in the comment. Krippendorff’s alpha coefficient [37] was then used to measure the interrater agreement between both evaluators. Krippendorff’s alpha coefficient is a reliability coefficient employed frequently in content analysis, developed to measure the agreement among codes that draw distinctions among typically unstructured phenomena. Acceptable inter-rater agreement is considered an alpha above or equal to 0.8 [37].

We then identified, through an inductive coding scheme, an emotional valence (positive, negative, or neutral) expressed by participants towards the quality of the care transitions they had experienced associated with each individual theme. We also calculated an interrater Krippendorff’s alpha reliability coefficient for the emotional valence codes. We then produced descriptive statistics for each theme stratified based on their emotional valence and by time period (before and after the start of the COVID-19 pandemic).

### Sample size

For the LEARNING WISDOM cohort, our sample size for the HDL site was calculated based on measuring a daily CTM-3 [38] for five discharged patients per day from the ED aged more than 65 years. The other three sites had a planned sample size of three patients per day from June 21st, 2019, until December 21st, 2021. After collecting data between January 24th, 2019, to October 4th, 2019, at the HDL site yielding an average score of 75.1 (95% CI [67.6, 82.6]), we decreased our recruitment of five random patients per day to three patients a day—like the other three sites—because including three patients per day until the end of recruitment would be sufficient to attain sufficiently precise daily CTM-3 point estimates.

For our nested qualitative study, we selected a sample of participants’ responses using a random number generator to obtain a representative sample of participants from all periods of the three-year study (pre and during COVID-19). Two authors (VC and NG) performed the qualitative inductive content analysis using the comments from sampled participants. We coded comments placed in an online spreadsheet until we reached data saturation when additional comments did not reveal new themes [39]. For each hospital site, two authors (VC and NG) each individually coded 30 randomly selected comments (selection without replacement). After which they each individually analyzed additional randomly selected comments in rounds of 10. We considered saturation achieved when two consecutive rounds of 10 comments were completed without the emergence of a new sub-theme per site.

### The impact of the COVID-19 pandemic

To analyze how the COVID-19 pandemic may have affected the experiences of older adult patients undergoing a transition of care, we split the coded comments into two groups: patients who left the ED before the onset of the COVID-19 pandemic, and patients who left the ED on or after the start of the pandemic on March 14th, 2020 [28].

## Results

**Fig. 1:**
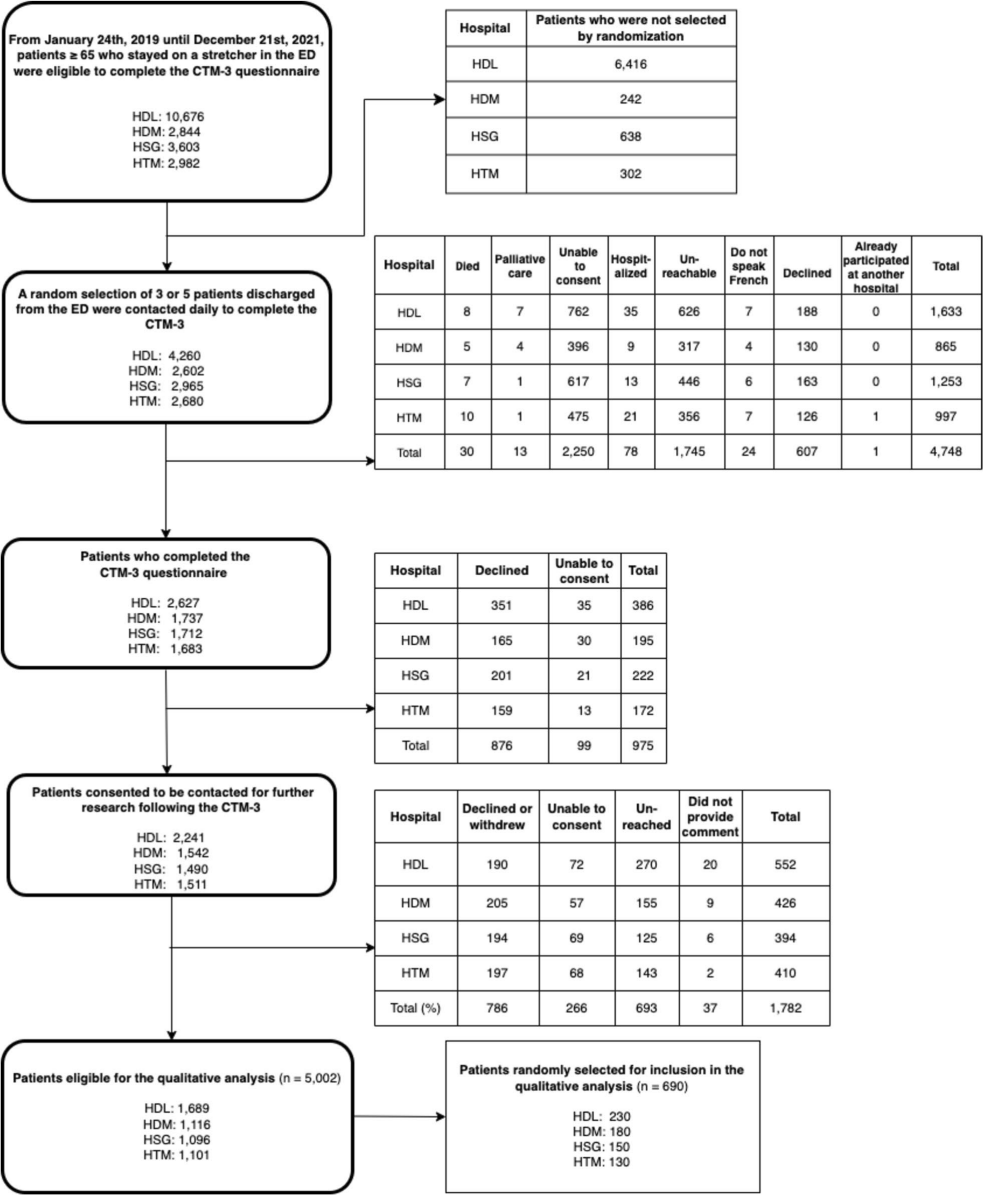
Flowchart of recruitment process

Figure 1 presents the flow of participants during our recruitment process. We reached data saturation after analyzing a sample of comments from 690 patients (339 pre-COVID and 351 during-COVID) composed of 351 women (50.9%) and 339 men (49.1%). Demographic characteristics of the sample are summarized in Table 1. Patients were aged 75 years on average (*M* = 75.7, *SD* = 7.2) and nearly all were Caucasian (99.9%) and spoke French as their first language (99.7%). We did not observe differences among patients included in this qualitative analysis (*N* = 690) versus all participants in the LEARNING WISDOM project who provided a comment but were not selected via random sampling (*N* = 4,312), and patients who opted not to provide a comment at all (*N* = 37). See Supplemental Table 2d in Appendix B. Included patients reported on average that 3.8 people (*SD* = 3.7) in their entourage could serve as sources of social support, and one third of patients reported having an informal caregiver (35.8%). Nearly all patients reported having access to transport for medical appointments (94.1%) and a family physician (91.6%), but only half of patients (51.7%) said they could quickly get an appointment with their family physician if needed. Patients were discharged from one of the four EDs before the COVID-19 pandemic (01/01/2019 to 12/03/2020) and throughout waves one (13/03/2020 to 11/07/2020), two (23/08/2020 to 20/03/2021), three (21/03/2021 to 17/07/2021), four (18/07/2021 to 4/12/2021) and five (5/12/2021 to 12/03/2022). Our sample included slightly more participants who were discharged from the ED after the start of the COVID-19 pandemic (*N* = 351, 50.8%) than before the start of the pandemic in Québec, Canada (*N* = 339, 49.1%). Concerning participants’ scores on the CTM-3, for each of the three questions, the majority of patients strongly agreed, or agreed with each statement. Additionally, the distribution of these responses did not statistically significantly change between the two time periods (see Table 1 and Appendix A). The mean CTM-3 scores were also both similarly high without any significant difference in both periods (see Table 1 and Appendix A).

**Table 1:** Demographic characteristics of randomly selected participants included in the qualitative analysis, stratified by pandemic period

	Before COVID-19 (***N***=339)	During COVID-19 (***N***=351)	Overall (***N***=690)
**Hospital**			
Hôtel-Dieu de Lévis	103 (30.4%)	127 (36.2%)	230 (33.3%)
Hôpital de Montmagny	91 (26.8%)	89 (25.4%)	180 (26.1%)
Hôpital de Saint-Georges	80 (23.6%)	70 (19.9%)	150 (21.7%)
Hôpital de Thetford-Mines	65 (19.2%)	65 (18.5%)	130 (18.8%)
**Age**			
Mean (SD)	76.3 (7.48)	75.2 (6.88)	75.7 (7.20)
**Sex**			
Men	177 (52.2%)	162 (46.2%)	339 (49.1%)
Women	162 (47.8%)	189 (53.8%)	351 (50.9%)
**Charlson Comorbidity Index score**			
Mean (SD)	5.08 (2.20)	4.68 (2.04)	4.88 (2.13)
**First language**			
French	339 (100%)	349 (99.4%)	688 (99.7%)
English	0 (0%)	2 (0.6%)	2 (0.3%)
**Ethnicity**			
Caucasian	339 (100%)	350 (99.7%)	689 (99.9%)
Missing	0 (0%)	1 (0.3%)	1 (0.1%)
**Annual income, CAD**			
< 10 000$	15 (4.4%)	1 (0.3%)	16 (2.3%)
10 000 - 19 999$	62 (18.3%)	41 (11.7%)	103 (14.9%)
20 000 - 29 999$	57 (16.8%)	63 (17.9%)	120 (17.4%)
30 000 - 39 999$	37 (10.9%)	53 (15.1%)	90 (13.0%)
40 000 - 49 999$	23 (6.8%)	39 (11.1%)	62 (9.0%)
50 000 - 59 999$	20 (5.9%)	14 (4.0%)	34 (4.9%)
60 000 - 69 999$	5 (1.5%)	11 (3.1%)	16 (2.3%)
70 000 - 79 999$	2 (0.6%)	7 (2.0%)	9 (1.3%)
80 000 - 89 999$	1 (0.3%)	6 (1.7%)	7 (1.0%)
90 000 - 99 999$	1 (0.3%)	7 (2.0%)	8 (1.2%)
> 100 000$	7 (2.1%)	8 (2.3%)	15 (2.2%)
Preferred not to respond	107 (31.6%)	78 (22.2%)	185 (26.8%)
Missing	2 (0.6%)	23 (6.6%)	25 (3.6%)
**Highest level of education**			
Primary school	138 (40.7%)	175 (49.9%)	313 (45.4%)
Secondary school	96 (28.3%)	82 (23.4%)	178 (25.8%)
Trade or professional school	28 (8.3%)	24 (6.8%)	52 (7.5%)
College degree	35 (10.3%)	40 (11.4%)	75 (10.9%)
Bachelor's degree	30 (8.8%)	21 (6.0%)	51 (7.4%)
Graduate degree	11 (3.2%)	9 (2.6%)	20 (2.9%)
Missing	1 (0.3%)	0 (0%)	1 (0.1%)
**Living situation**			
Home	191 (56.3%)	203 (57.8%)	394 (57.1%)
Home, alone	97 (28.6%)	101 (28.8%)	198 (28.7%)
Nursing home, 24h access to nurse	27 (8.0%)	27 (7.7%)	54 (7.8%)
Nursing home, no nurse on site	14 (4.1%)	13 (3.7%)	27 (3.9%)
Affordable housing	0 (0%)	0 (0%)	0 (0%)
Family Type Ressources (FTR)	0 (0%)	0 (0%)	0 (0%)
Long term care residence	0 (0%)	0 (0%)	0 (0%)
Other	0 (0%)	0 (0%)	0 (0%)
Missing	10 (2.9%)	7 (2.0%)	17 (2.5%)
**Size of social network (persons)**			
Mean (SD)	3.42 (3.96)	4.14 (3.48)	3.79 (3.74)
**Have a family physician**			
Yes	316 (93.2%)	316 (90.0%)	632 (91.6%)
No	23 (6.8%)	35 (10.0%)	58 (8.4%)
**Able to quickly see their family physician**			
Yes	198 (58.4%)	159 (45.3%)	357 (51.7%)
No	113 (33.3%)	145 (41.3%)	258 (37.4%)
Unsure	12 (3.5%)	12 (3.4%)	24 (3.5%)
Missing	16 (4.7%)	35 (10.0%)	51 (7.4%)
**Have access to transport**			
Yes	313 (92.3%)	336 (95.7%)	649 (94.1%)
No	26 (7.7%)	15 (4.3%)	41 (5.9%)
**Have a caregiver (self-report)**			
No	207 (61.1%)	236 (67.2%)	443 (64.2%)
Yes	132 (38.9%)	115 (32.8%)	247 (35.8%)
**Care Transitions Measure – 3**			
**The hospital staff took my preferences and those of my family or caregiver into account in deciding what my health care needs would be when I left the hospital**			
Strongly agree	129 (38.1%)	183 (52.1%)	312 (45.2%)
Agree	108 (31.9%)	123 (35.0%)	231 (33.5%)
Disagree	17 (5.0%)	17 (4.8%)	34 (4.9%)
Strongly disagree	6 (1.8%)	7 (2.0%)	13 (1.9%)
Missing	79 (23.3%)	21 (6.0%)	100 (14.5%)
**When I left the hospital, I had a good understanding of the things I was responsible for in managing my health**			
Strongly agree	167 (49.3%)	164 (46.7%)	331 (48.0%)
Agree	126 (37.2%)	121 (34.5%)	247 (35.8%)
Disagree	28 (8.3%)	42 (12.0%)	70 (10.1%)
Strongly disagree	7 (2.1%)	7 (2.0%)	14 (2.0%)
Missing	11 (3.2%)	17 (4.8%)	28 (4.1%)
**When I left the hospital, I clearly understood the purpose for taking each of my medications**			
Strongly agree	164 (48.4%)	196 (55.8%)	360 (52.2%)
Agree	92 (27.1%)	96 (27.4%)	188 (27.2%)
Disagree	21 (6.2%)	22 (6.3%)	43 (6.2%)
Strongly disagree	6 (1.8%)	2 (0.6%)	8 (1.2%)
Missing	56 (16.5%)	35 (10.0%)	91 (13.2%)
**Transformed (linear) Care Transition Measure - 3 Score**			
Mean (SD)	84.8 (15.3)	85.9 (25.2)	85.4 (15.2)

### Main themes and their sub-themes

**Fig. 2:**
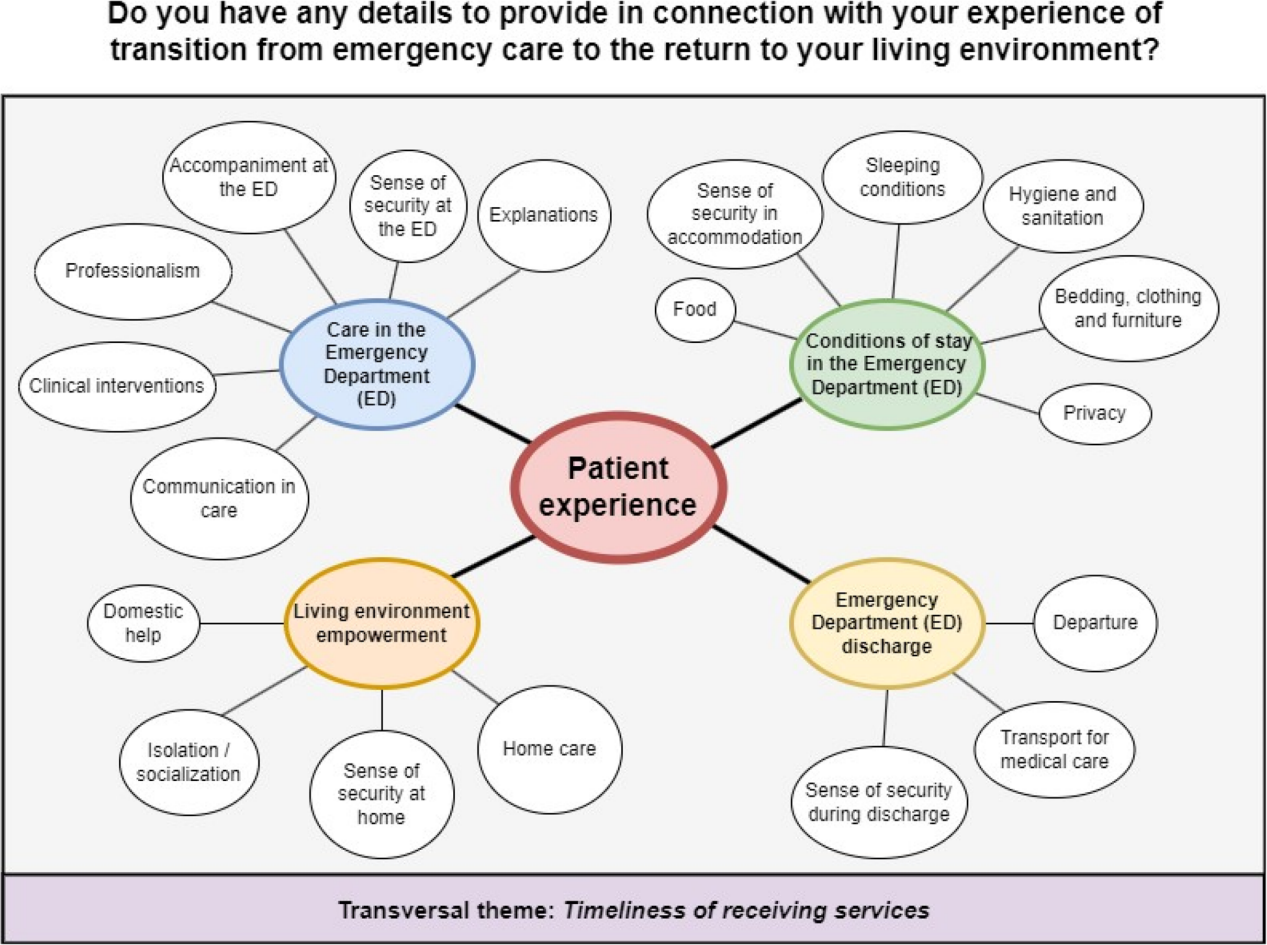
*Mind Map* of the four main themes and nineteen sub-themes emerging from patients’ comments about their complete care transition continuum experience before, during and after their ED visit and discharge back to their living situations. A transversal theme, *Timeliness of receiving services* is also identified

We identified four overarching themes related to transitions of care among older adult ED users, and eighteen sub-themes. Both coders obtained a Krippendorff’s alpha coefficient of 0.93 on the first round of coding, and after a discussion to resolve disagreements in codes we achieved an alpha of 0.98. These main themes were (1) care in the ED, (2) conditions of stay in the ED, (3) leaving the ED, and (4) empowering patients in their living environment. A *Mind Map* of all themes and their sub-themes is presented in Fig. 2.

#### Care in the ED

**Fig. 3:**
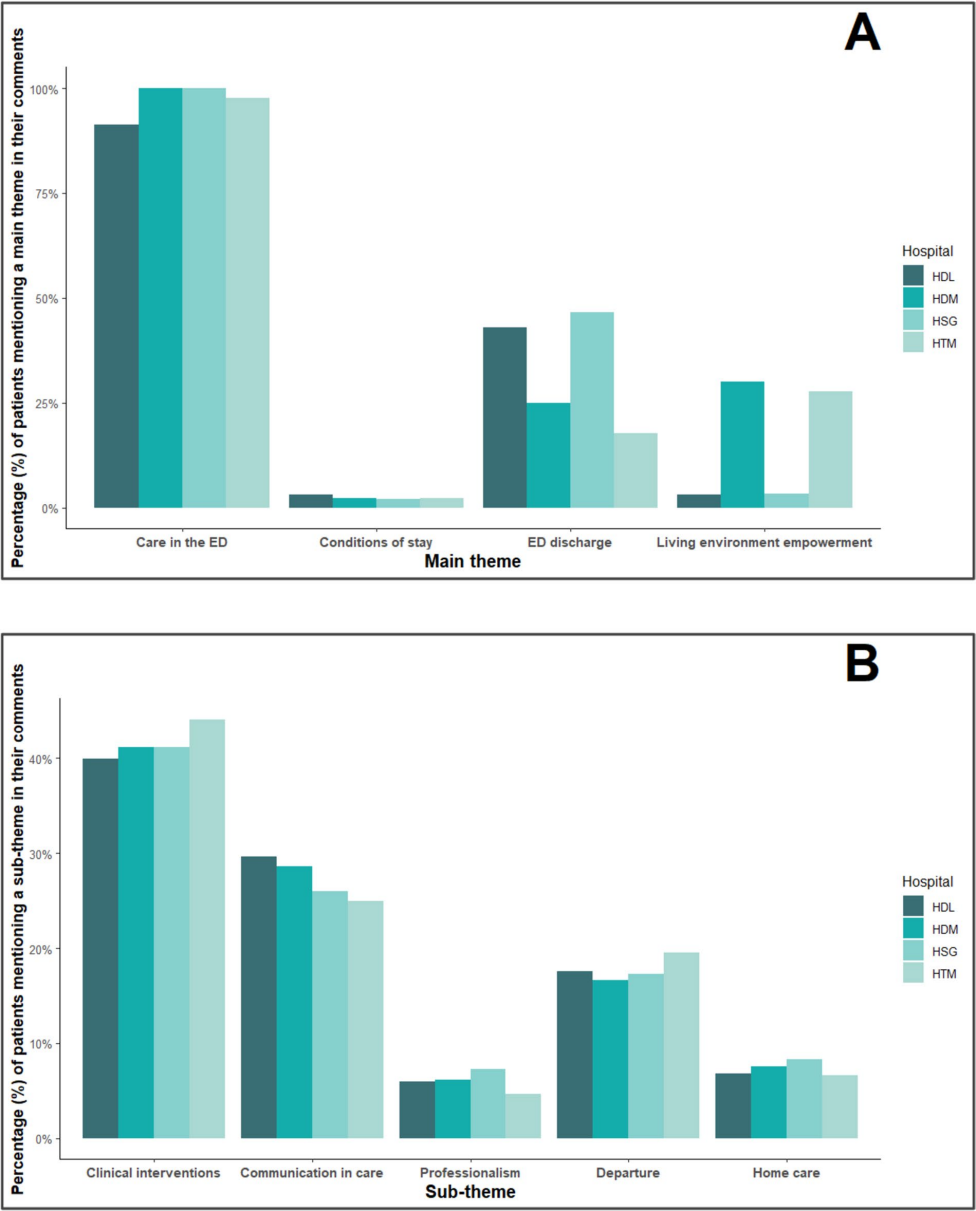
**A** Barplot of the relative frequency (%, *y*-axis) of main themes (*x*-axis) coded in patient comments about their care transition. **B** Barplot of the relative frequency (%, *y*-axis) of the five most common sub-themes (*x*-axis). The legend to the right of each plot identifies the hospital. For each bar in the plots in both figures, the *numerator* is equal to the number of patients that mentioned a theme or sub-theme in their comment and the *denominator* is equal to the total number of patients who provided a comment, at a given hospital

Quality of care was the most frequently cited main theme by patients, present in 96.6% of all comments (Fig. 3A).

This theme was related to several sub-themes: clinical interventions (cited in 79.4% of all patient comments, see Fig. 3B), communication (53.2%), professionalism (11.6%), explanations (5.2%), sense of security about care (2.3%) and finally accompaniment (1%). Clinical intervention comments reflected the quality of acute care. Communication consisted of comments citing the presence or absence of planned follow-ups or tests with specialists or outpatient clinics. For professionalism, patients reported whether the ED staff members were negligent, empathetic, dismissive, prejudiced, or did not take their complaints seriously. In the case of explanations, patients reported whether personnel took the time to meaningfully explain planned procedures and tests in plain language. Security encompassed feelings of safety and the preservation of patients’ own bodily autonomy during care. Lastly, accompaniment at the ED included comments about loved ones who stayed with and supported the patient during their ED stay.

#### Conditions of stay in the ED

The conditions of stay on a stretcher were the least mentioned of the main themes (2.4% of all patient comments, see Fig. 3A). Sub-themes included food, sense of security, sleeping conditions, hygiene and sanitation, privacy, and bedding (each < 1%, see Fig. 3B). For food, the commonest concerns were quality and the availability of drinking water. Sense of security about the environment referred to feeling safe during their observation in the physical ED environment. Sleeping conditions highlighted excessive light which made it difficult to rest. For hygiene and sanitation, the concerns were oriented toward inadequate cleanliness of facilities and observed inadequacies in personal protective equipment used by staff. Privacy reflected the ability to discreetly speak to staff and to attend to private matters with dignity (e.g., toileting). For bedding, patients mentioned uncomfortable furniture, bedding, bedclothes, or accommodations and whether their environment was adequately heated or cooled.

#### ED discharge

ED discharge was mentioned in approximately 34.3% of all main theme comments, making it the second most cited main theme (see Fig. 3A). Comments described the circumstances surrounding the conclusion of ED care and the return home. This main theme was divided into three sub-themes: departure (33.9%), the presence or absence of transport, and feelings of security or vulnerability in leaving the ED (each < 1.3%, see Fig. 3B). Here, patients mentioned whether their ED discharge was seamless, rushed or forced, at night, too slow, or if the ED had to close temporarily because the ED physician had to escort a patient during an urgent medical transfer to another hospital forcing some patients to leave consequently. Although this is a very rare situation, some smaller remote community-based hospitals have staffing issues that force them to have to close temporarily if the on-call physician needs to escort a patient in ambulance for a life-saving procedure in another hospital.

#### Living environment empowerment

The main theme related to services empowering older adults to stay independent at home was specified in 14.8% of all patient comments (Fig. 3A). This main theme was subdivided in the following sub-themes: home care (14.1%), domestic help, isolation, and sense of security at home (each < 1%) (Fig. 3B). Concerning home care, patients commented on interventions or services performed outside the hospital which required a specific level of training. Domestic help referred to having a person, a relative, or services for daily chores and household tasks. As for isolation, the comments concerned the potential social isolation due to patients’ absence or small social networks. The sense of security at home encompassed comments from patients about their sense of feeling safe and secure in their living environment.

Main themes were reported with similar frequency regardless of the hospital site analysed, despite differences in hospital size and the annual visit rate among the four sites (Fig. 3A). The most frequently mentioned sub-themes (mentioned > 90% of the time) for each site (Fig. 3B) were (i) clinical interventions, (ii) communications between departments, (iii) professionalism, (iv) ED discharge and (v) home care. These themes represent the most salient topics of concern among older adults undergoing care transitions. For the remaining sub-themes, we noted only anecdotal differences (< 3%) among frequencies observed between sites.

#### Timeliness of services in and after the ED

The timeliness of receiving services in and after ED care was a transversal theme affecting all our main themes and subthemes. We did not identify the timeliness of services as an individual sub-theme in each of our major themes as it affected each theme individually. For Care in the ED, time was mentioned by 76 patients (100% of comments referenced wait times) affecting all 6 related subthemes (clinical interventions, explanations, security in the ED, communications between departments, professionalism, and accompaniment). For Living environment empowerment, time was mentioned by 14 patients, affecting 3 subthemes (home care, domestic help, and isolation. e.g., waiting list for domestic or home care services). For Conditions of stay in the ED, time was mentioned by 3 patients and concerned the time to receive a service in the ED, referring to 3 subthemes (sleeping conditions, food, and hygiene, e.g., spending the night on an uncomfortable gurney in a bright hallway, waiting to have soiled protective underwear changed, and time spent without eating). For ED discharge, time was mentioned by 26 patients and concerned all 3 subthemes (departure and whether patients felt discharge was too soon or took too long, delays in receiving transportation home, and sense of security waiting to be discharged). For examples and full comments, see Appendix C.

### Emotional valence

**Fig. 4:**
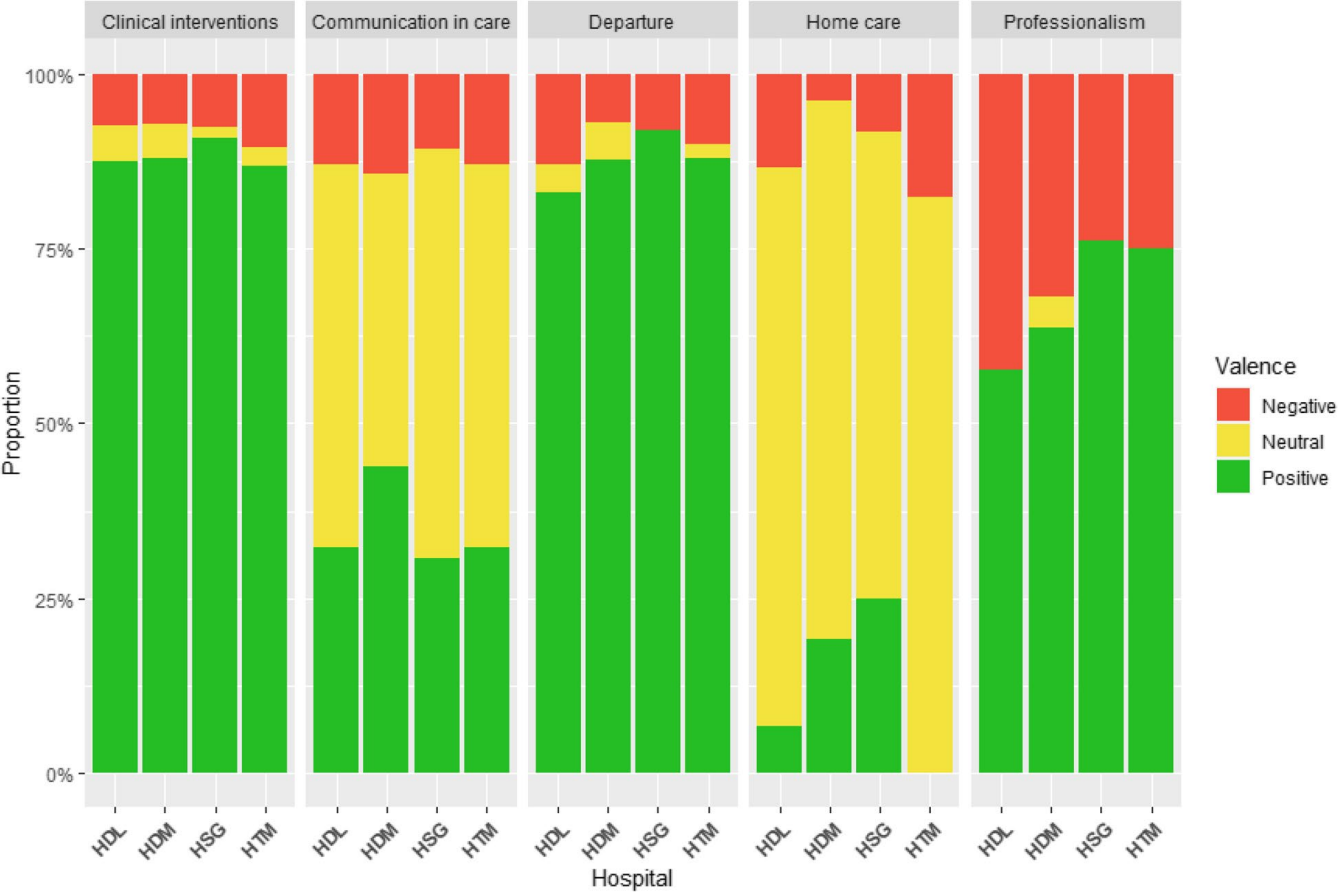
Histogram representing satisfaction (%, y-axis) regarding each sub-theme. Each bar denotes the proportion of negative, positive, and neutral comments that emerged among all comments mentioning the given sub-theme. Sub-themes are presented in decreasing frequency from left to right, and sub-themes were analysed for each site separately. Only the top five most frequent sub-themes are presented. Both time periods (before the COVID-19 pandemic and during the COVID-19 pandemic) were collapsed together

We determined an indication of the patient’s sense of emotional valence (positivity, negativity, or neutrality) associated with each comment using an inductive approach. Interrater reliability was 0.94 using Krippendorff’s alpha for this coding of emotional valence. This emotional valence was mostly influenced by patients’ assessment of the quality of care, services received, and their timeliness. The emotional valence was positive among 81% of the comments in the cohort and positive in 76% of comments mentioning wait times. There were 70 comments that mentioned timeliness of care: 58 were positive, 11 negative and 7 neutral. Among the 11 negative comments, 10 reported long wait times. Because all four hospital sites had the same top sub-themes, we compared them on the emotional valence calculated by the number of positive, neutral, and negative comments (see Fig. 4). Overall, the clinical interventions and departure were the most positively described sub-themes, whereas communication in care and professionalism were the most frequently mentioned sub-theme with a negative valence. Home care services were the services that received neutral comments the most frequently and positive comments least frequently.

### Effects of the COVID-19 pandemic

The proportion of positive comments relating to communication in care before the pandemic decreased in favor of more neutral comments after the start of the pandemic (14.2% increase in neutral comments, and 26.8% reduction in positive comments). Participants appeared to be more pleased with clinical care during the pandemic than before the pandemic (26.4% increase in positive comments), as well as their experiences with departing the ED (13.8% increase in positive comments). Chi-square tests of proportions of the effect of *Valence ✕ Time Period* yielded statistically significant effects of emotional valence as a function of the COVID-19 pandemic for all five themes. The proportions of emotional valences before and during the pandemic are presented in Table 2.

**Table 2:** Emotional valence frequencies for the top five sub-themes before and after the start of the COVID-19 pandemic. Percentages represent the number of comments for each case divided by the total number of comments in the time period. Values with * represent values from a Yates corrected Chi-square (appropriate when cell values ≤ 5). “No response” represents comments for which a given theme did not emerge

Sub-theme	Before the onset of the pandemic (***n*** = 339)	After the onset of the pandemic (***n*** = 351)	***X*** ^**2**^ test
**Clinical interventions**	Negative: 25 [7.3%]	Negative: 13 [3.7%]	58.54 *p* < .001
	Neutral: 11 [3.2%]	Neutral: 10 [2.8%]	
	Positive: 189 [55.7%]	Positive: 288 [82.1%]	
	No response: 114 [33.6%]	No response: 41 [11.6%]	
**Communication in care**	Negative: 25 [7.4%]	Negative: 23 [6.6%]	111.46 *p* < .001
	Neutral: 70 [20.6%]	Neutral: 122 [34.8%]	
	Positive: 108 [31.9%]	Positive: 18 [5.1%]	
	No response: 86 [25.3%]	No response: 188 [53.5%]	
**Departure**	Negative: 15 [4.4%]	Negative: 6 [17.1%]	12.84* *p* = .004
	Neutral: 3 [0.9%]	Neutral: 4 [1.1%]	
	Positive: 76 [22.4%]	Positive: 127 [36.2%]	
	No response: 245 [72.2%]	No response: 251 [71.5%]	
**Home care**	Negative: 5 [1.5%]	Negative: 5 [1.4%]	22.48* *p* < .001
	Neutral: 17 [5%]	Neutral: 57 [16.2%]	
	Positive: 7 [2.5%]	Positive: 6 [1.7%]	
	No response: 310 [91.4%]	No response: 283 [80.6%]	
**Professionalism**	Negative: 13 [3.8%]	Negative: 12 [3.4%]	8.06* *p* = .044
	Neutral: 0	Neutral: 1 [0.3%]	
	Positive: 37 [10.9%]	Positive: 17 [4.8%]	
	No response: 289 [85.2%]	No response: 321 [91.4%]	

## Discussion

We sought to generate a better understanding of patient’s care transition experiences that could be used in designing future patient-oriented interventions to improve care transitions in the post-COVID-19 context. We used open-ended comments to collect the perspectives of older adults’ experiences in the ED and their subsequent care transition from the ED. We conducted a qualitative content analysis using an inductive approach to identify themes and sub-themes among these comments to record concerns of patients from four Québec hospitals. We obtained a high level of agreement between coders and a clear coding scheme [40]. We also noted the emotional valence of each theme and sub-theme embedded within patients’ comments to understand if they were satisfied or not about the care transitions they experienced.

For each site, the most frequently mentioned sub-themes (mentioned over 90% of the time) were the clinical interventions received in the ED, communications in care, professionalism, discharge from the ED, and home care. These sub-themes represent the most salient topics of concern among older adults undergoing care transitions. Overall, quality of care was reported positively but communication in care and home care were qualified as neutral. These results are similar to findings by other authors [15, 41, 42] who report that older adults who undergo transitions in care also mention the quality of care received in hospital, but that communication during ED discharge and care transition planning can be fragmented. These themes will be important to consider in designing future patient-oriented interventions to improve care transitions in the post-COVID-19 context.

In home care, the emotional valence was mostly neutral, believed to be because a service had been organized in the ED but had not yet been delivered. As such, patients could only comment on the fact that a service was to come, but not on the quality or experience of receiving that service. The proportion of positive comments relating to communication in care before the pandemic decreased in favor of more neutral comments after the start of the pandemic. This is likely because of the service slowdown hospitals experienced at the beginning of the COVID-19 pandemic [43].

Similar results were found in qualitative analyses of patient and caregiver experiences, such that transport, communication, and services at home were commonly cited themes [44]. Lapses in communication were argued to be a major barrier to successful care transitions [44, 45]. Like other authors, we have also found that patients report an overall positive perception of acute care services [46]. The COVID-19 pandemic appears to have significantly impacted patient comments in our study. In a qualitative study on care transition experiences during the COVID-19 pandemic, communication was often perceived negatively, whereas in our study communication was often addressed neutrally [47].

We also observed that communications in care and professionalism at the ED are often negatively perceived and may be important barriers to successful care transitions among older adults. When patients are asked in surveys to score their experience in the ED, other authors have found that their answers are misquoted as they attribute positive scores regarding certain aspects they also expressed negative comments about [48]. These surveys may also inadvertently influence patients’ answers which either misrepresent some aspects or overrepresent some they would otherwise not have addressed. A deeper understanding of patients’ experiences could better guide patient-centered quality improvement initiatives compared to only using quantitative satisfaction scores (e.g., CTM-3). Future work using natural language processing (NLP) [49] could also potentially offer new opportunities to analyse patient-reported qualitative comments using patients’ own vocabulary to qualify their care transitions.

Orchestrating cooperation among health workers increases patient satisfaction [50] and reduces length of hospital stay [51]. If we can ensure that older adult patients are satisfied with their care, we may reap the rewards of fewer avoidable visits to the ED [52]. The benefits of a successful care transition are not limited to individual patients as they impact on communities and caregivers. The World Health Organization’s Decade of Health Aging Report considers this a crucial holistic indicator of care transition success [53].

Patients who are satisfied with their care and feel a sense of self-efficacy tend to stay out of the hospital longer and have more favorable health outcomes [54, 55]. Even so, the causal link between discharge planning, patient satisfaction, and hospital-level outcomes is still unclear [55]. When older adults feel empowered to manage their health at home, they show greater adherence to treatment plans [56]. It stands to reason that effective care transitions must provide clear instructions and resources to empower older adults to ensure a complete transition.

The American Geriatrics Society argues that its own guidelines for an ideal geriatric ED should be tailored to each ED based upon patient needs and available resources [57]. We found that patients appear to be satisfied overall with the acute interventions they receive in the four EDs we studied, but patients report neutral and negative comments relating to communication between the acute care provider and external clinics and specialists, their departure from the ED to their living environment, and the professionalism of the staff at the ED. All these elements would help the four EDs participating in this study to develop tailored interventions aiming to improve their local care transition best practices. In addition to using validated quantitative questionnaires such as the CTM-3 to measure quality of care transitions, our study also points to the usefulness of collecting open ended qualitative data from patients after their discharge to gain additional insights to inform a model for a care transition quality improvement initiative in other EDs. For our part, the comments collected and the results from this analysis will help in the design and implementation of a patient-oriented intervention to improve care transitions at the CISSS-CA immediately and in the post-COVID-19 era.

Overall, our results indicate that all the major sub-themes that emerged in our data appear to have been influenced by COVID-19. Most notable were changes in the proportion of positive comments relating to communication in care, which shifted in tone from positive to neutral. Future pandemic mitigation strategies will have to focus on improving communication throughout the care continuum (referrals, communications between clinics or specialties, patient and caregiver empowerment), and the preservation of homecare services. Among potential patient-oriented solutions that need further investigation would be better patient-centered communication skills among health professionals [58], addressing new patient decisional needs earlier [59, 60], decreasing loneliness and social isolation [61], having a single electronic medical record shared across different care settings [62] and accessible to patients themselves [63], better virtual care services and tools [14], and more efficient knowledge mobilization and management [64].

### Strengths and limitations

The strengths of our study arise from our rigorous application of qualitative methodology, the strong inter-rater agreement, and the substantial random sample size that we recruited representing patients seen pre and during COVID-19 pandemic periods at four different EDs. Our strong inter-rater reliability indicates a clear coding scheme, which we attribute to a rigorous, iterative development of the coding scheme. Our large sample size can be explained by our desire to attain saturation of themes and sub-themes at all four sites.

This study also has limitations. First is the short follow-up time after departure from the ED as patients were called to participate between 1 and 7 days following discharge. Questions were fielded as soon as possible following this post-discharge period to capitalize on the primacy of the patient’s experience. While this primacy may have been a strength, this short follow-up time may not have left sufficient time for the patient to undergo all relevant aspects of the care transition, leading to an information bias. For instance, many patients reported a planned follow-up with a specialist but had yet to attend, so this experience was coded neutrally.

Second, since all our patients were recruited after a stay in the observation unit, we may have selected patients with short door-to-first contact wait times. This may have underestimated the impact of wait times on patient’s satisfaction with care because all patients were triaged to stretchers in the ED observation unit, and not strictly ambulatory where less urgent cases wait longer to see a doctor. As such, wait times to first contact with a nurse and doctor may have been shorter than ambulatory patients. However, this is not absolute because when stretchers were full, patients remained in the waiting room [65] and overcrowding can negatively influence total length of stay in the ED. In future work, door-to-doctor time (from when a patient arrives at the ED to their first personal interaction with a doctor) could be used to corroborate patient experiences, as this metric is significantly associated with worse patient experience in discharged patients [66].

Third, a selection bias may have occurred due to the utilization of the telephone to administer our questionnaire, limiting the participation of seniors who hear less well, those who do not use the telephone, and those who do not possess a telephone. However, this also allowed us to survey patients with reduced mobility. We also note a potential social desirability bias as patients satisfied with their care may have been more willing to participate, and dissatisfied patients may have declined due to a perceived risk that they may harm their relationship with their health professional. This potential bias did not prevent us from collecting negative comments and areas for improvement.

Fourth, patient comments for this study were collected over the telephone and transcribed immediately by a research professional. We did not audio record patients’ comments. This may have introduced an information bias such that the content of comments has been interpreted by the research professional conducting the interviews. Also, patients may not have completely understood our question about care transitions and answered our questions with their overall care in mind. This might reflect itself in the themes we identified that covered many aspects of quality of care within the ED and not specific to care transitions between the ED and the community. Although this may not have been clear for patients, we also believe that including feedback from patients about the complete care transition continuum before, during and after their ED visit is crucial because poor care transitions can find their root cause in the lack of meaningful advance care planning, iatrogenic complications related to ED conditions of stay, substandard communication with caregivers during ED stay, and deficient care coordination post ED discharge. Our collection method does not appear to have influenced the length of the comments collected, as ranged from a few words up to 100 words of text. We also took care to transcribe local metaphors and idioms provided by patients to emphasize the depth of their feelings. One man described feeling like “*un chien dans un jeu de quilles*” [“*a dog in a bowling alley” analogous to feeling like you are out of place*] when he arrived at the ED for care, indicating a strong feeling of being misunderstood and unwelcome. For more examples of each theme, their translations, and definitions, see Appendix C.

## Conclusion

The present study offers a holistic understanding of individual experiences at the emergency department among older adult patients. Overall, patients were satisfied by their care, but they also reported neutral and negative comments relating to communication between the acute care provider and external clinics, specialists and community services, their departure from the hospital to their living environment, and the professionalism of the staff in the emergency department. The themes identified will help design patient-oriented interventions focused on improving the quality-of-care transitions from emergency departments for older adults.

### Supplementary Information

**Additional file 1.**

## Data Availability

The datasets created and analysed during the current study are available from the corresponding author on reasonable request.
