# Supplementary material for: Transitions of care for older adults discharged home from the emergency department: an inductive thematic content analysis of patient comments
Source: BMC Geriatr. 2024 Jan 3;24:8. doi: 10.1186/s12877-023-04482-0 (PMC10763115; doi:10.1186/s12877-023-04482-0)
Supplement: Supplementary file 1 — Additional file 1. [file 12877_2023_4482_MOESM1_ESM.docx]

**Supplementary Materials**

Appendix A

**Three-Item Care Transition Measure – CTM-3**

| Item number | Question (English) | Question (French) | Response options |
| --- | --- | --- | --- |
| 1 | The hospital staff took my preferences and those of my family or caregiver into account in deciding what my health care needs would be when I left the hospital. | *Le personnel de l'hôpital a tenu compte de ce que je souhaitais pour mes soins de santé à ma sortie de l'hôpital.* | Strongly agree  Agree  Disagree  Strongly Disagree  Missing/Don’t Know/Don’t Remember/Not Applicable |
| 2 | When I left the hospital, I had a good understanding of the things I was responsible for in managing my health. | *À ma sortie de l'hôpital, j'avais compris de manière claire comment prendre en charge ma santé.* | Strongly agree  Agree  Disagree  Strongly Disagree  Missing/Don’t Know/Don’t Remember/Not Applicable |
| 3 | When I left the hospital, I clearly understood the purpose for taking each of my medications. | *Lorsque j'ai quitté l'hôpital, je comprenais parfaitement pourquoi je devais prendre chacun de mes médicaments.* | Strongly agree  Agree  Disagree  Strongly Disagree  Missing/Don’t Know/Don’t Remember/Not Applicable |

**Analyses of the** **Three-Item Care Transition Measure – CTM-3**

| **Care Transitions Measure – 3** | | | |  |
| --- | --- | --- | --- | --- |
|  | **Before**  **COVID-19   (N=339)** | **During**  **COVID-19   (N=351)** | **Overall  (N=690)** | **Statistical test of the effect of time period (before or during**  **COVID-19)** |
| **The hospital staff took my preferences and those of my family or caregiver into account in deciding what my health care needs would be when I left the hospital.** |  |  |  | *X*^2^ = 2.12, *p* = .548 |
| Strongly agree | 129 (38.1%) | 183 (52.1%) | 312 (45.2%) |  |
| Agree | 108 (31.9%) | 123 (35.0%) | 231 (33.5%) |  |
| Disagree | 17 (5.0%) | 17 (4.8%) | 34 (4.9%) |  |
| Strongly disagree | 6 (1.8%) | 7 (2.0%) | 13 (1.9%) |  |
| Missing | 79 (23.3%) | 21 (6.0%) | 100 (14.5%) |  |
| **When I left the hospital, I had a good understanding of the things I was responsible for in managing my health.** |  |  |  | *X*^2^ = 2.87, *p* = .411 |
| Strongly agree | 167 (49.3%) | 164 (46.7%) | 331 (48.0%) |  |
| Agree | 126 (37.2%) | 121 (34.5%) | 247 (35.8%) |  |
| Disagree | 28 (8.3%) | 42 (12.0%) | 70 (10.1%) |  |
| Strongly disagree | 7 (2.1%) | 7 (2.0%) | 14 (2.0%) |  |
| Missing | 11 (3.2%) | 17 (4.8%) | 28 (4.1%) |  |
| **When I left the hospital, I clearly understood the purpose for taking each of my medications.** |  |  |  | *X*^2^ = 3.14, *p* = .369 |
| Strongly agree | 164 (48.4%) | 196 (55.8%) | 360 (52.2%) |  |
| Agree | 92 (27.1%) | 96 (27.4%) | 188 (27.2%) |  |
| Disagree | 21 (6.2%) | 22 (6.3%) | 43 (6.2%) |  |
| Strongly disagree | 6 (1.8%) | 2 (0.6%) | 8 (1.2%) |  |
| Missing | 56 (16.5%) | 35 (10.0%) | 91 (13.2%) |  |
| **Transformed (linear) CTM-3 Score** |  |  |  | *t*(688) = 0.99, *p* = .325 |
| Mean (SD) | 84.8 (15.3) | 85.9 (25.2) | 85.4 (15.2) |  |

*Note that values labelled as “Missing” were not included in the *X*^2^ analyses and represent cases where patients did not wish to answer or responded “I do not know” to the question.

**Open ended question:** Do you have any details to provide in connection with your experience of transition from emergency care to the return to your living environment? [*Avez-vous des précisions à apporter en lien avec votre expérience de transition de soins des urgences vers le retour à votre milieu de vie ?*]

Appendix B

**Supplementary tables.**

**Table 2a.** Emotional valence frequencies for the top five sub-themes before and after the start of the COVID-19 pandemic. Percentages represent the number of comments for each case divided by the total number of comments in the time period. Values with * represent values from a Yates corrected Chi-square (appropriate when cell values ≤ 5).

|  | **Before the onset of the pandemic (*n* = 339)** | **After the onset of the pandemic (*n* = 351)** | ***X*^2^ test** | ***Z-test (binomial test)*** |
| --- | --- | --- | --- | --- |
| **Clinical intervention** | Negative: 25 [7.3%]  Neutral: 11 [3.2%]  Positive: 189 [55.7%] | Negative: 13 [3.7%]  Neutral: 10 [2.8%]  Positive: 288 [82.1%] | 10.87  *p* = .004 | *p^neg^* = .037  *p^neu^* = .5  *p^pos^* < .001 |
| **Communication in care** | Negative: 25 [7.4%]  Neutral: 70 [20.6%]  Positive: 108 [31.9%] | Negative: 23 [6.6%]  Neutral: 122 [34.8%]  Positive: 18 [5.1%] | 74.97  *p* < .001 | *p^neg^* = .385  *p^neu^* < .001  *p^pos^* < .001 |
| **Departure** | Negative: 15 [4.4%]  Neutral: 3 [0.9%]  Positive: 76 [22.4%] | Negative: 6 [17.1%]  Neutral: 4 [1.1%]  Positive: 127 [36.2%] | 7.83*  *p* = .002 | *p^neg^* = .04  *p^neu^* = NA  *p^pos^* < .001 |
| **Home care** | Negative: 5 [1.5%]  Neutral: 17 [5%]  Positive: 7 [2.5%] | Negative: 5 [1.4%]  Neutral: 57 [16.2%]  Positive: 6 [1.7%] | 4.97*  *p* = .083 | *p^neg^* = NA  *p^neu^* < .001  *p^pos^* = NA |
| **Professionalism** | Negative: 13 [3.8%]  Neutral: 0  Positive: 37 [10.9%] | Negative: 12 [3.4%]  Neutral: 1 [0.3%]  Positive: 17 [4.8%] | 1.44*  *p* = .488 | *p^neg^* = .05  *p^neu^* = NA  *p^pos^* = .004 |

**Table 2b.** Mean [95% confidence interval] of linear-transformed scores on the three-item Care Transitions Measure (CTM-3) by hospital and sex.

|  | **Men** | **Women** | **Total** | ***t statistic*** | ***p value*** |
| --- | --- | --- | --- | --- | --- |
| **HDL** | 80.0 [75.9, 84.0] | 76.6 [72.7, 80.6] | 78.3 [75.4, 81.1] | -1.16 | .247 |
| **HDM** | 83.8 [80.0, 87.7] | 81.3 [76.7, 85.8] | 82.6 [79.6, 85.5] | -0.85 | .396 |
| **HSG** | 83.0 [79.1, 87.0] | 81.9 [77.6, 86.1] | 82.4 [79.6, 85.3] | -0.39 | .695 |
| **HTM** | 78.8 [73.8, 83.9] | 78.8 [73.0, 84.5] | 78.8 [75.0, 82.6] | -0.02 | .987 |
| **All sites** | 81.4 [79.4, 83.5] | 79.4 [77.1, 81.6] | 80.4 [78.9, 81.9] | -1.33 | .185 |

**HDL: Hôtel-Dieu de Lévis, HDM : Hôpital de Montmagny; HSG : Hôpital de Saint-Georges; HTM : Hôpital de Thetford-Mines**

**Table 2c.** Mean and [95% confidence interval] of linear-transformed scores on the care transitions measure (CTM-3) by hospital and time period.

|  | **Before COVID-19** | **During COVID-19** | ***t statistic*** | ***p value*** |
| --- | --- | --- | --- | --- |
| **HDL** | 77.0 [72.6, 81.3] | 79.3 [75.6, 83.0] | -0.81 | .419 |
| **HDM** | 81.6 [77.2, 86.0] | 83.6 [79.6, 87.6] | -0.68 | .5 |
| **HSG** | 80.8 [77.0, 84.6] | 84.3 [79.9, 88.7] | -1.18 | .239 |
| **HTM** | 79.0 [73.5, 84.4] | 78.6 [73.2, 84.1] | 0.08 | .929 |
| **All sites** | 79.5 [77.3, 81.7] | 81.3 [79.1, 83.4] | -1.13 | .261 |

**HDL: Hôtel-Dieu de Lévis, HDM : Hôpital de Montmagny; HSG : Hôpital de Saint-Georges; HTM : Hôpital de Thetford-Mines**

**Table 2d.** Demographic characteristics of patients included in the analysis, patients who were not selected via randomization, and patients who left no comment.

|  | **Included (N=690)** | **Not selected (N=4312)** | **Have no comment (N=37)** |
| --- | --- | --- | --- |
| **Hospital** |  |  |  |
| HDL | 230 (33.3%) | 1459 (33.8%) | 20 (54.1%) |
| HDM | 180 (26.1%) | 936 (21.7%) | 9 (24.3%) |
| HSG | 150 (21.7%) | 946 (21.9%) | 6 (16.2%) |
| HTM | 130 (18.8%) | 971 (22.5%) | 2 (5.4%) |
| **Sex** |  |  |  |
| Women | 351 (50.9%) | 2364 (54.8%) | 18 (48.6%) |
| Men | 339 (49.1%) | 1948 (45.2%) | 19 (51.4%) |
| **Age group** |  |  |  |
| 60 to 69 | 152 (22.0%) | 1105 (25.6%) | 4 (10.8%) |
| 70 to 79 | 348 (50.4%) | 2028 (47.0%) | 21 (56.8%) |
| 80 and up | 190 (27.5%) | 1179 (27.3%) | 12 (32.4%) |
| **Charlson Score** |  |  |  |
| Mean (SD) | 4.90 (2.13) | 4.83 (2.14) | 5.54 (2.05) |
| Median [Min, Max] | 4.00 [2.00, 15.0] | 4.00 [2.00, 14.0] | 5.00 [3.00, 11.0] |
| **No. of Comorbidities** |  |  |  |
| Mean (SD) | 1.84 (1.92) | 1.82 (1.91) | 2.32 (1.93) |
| Median [Min, Max] | 1.00 [0, 11.0] | 1.00 [0, 12.0] | 2.00 [0, 8.00] |
| **Infarcts** |  |  |  |
| No | 564 (81.7%) | 3634 (84.3%) | 31 (83.8%) |
| Yes | 126 (18.3%) | 676 (15.7%) | 6 (16.2%) |
| Missing | 0 (0%) | 2 (0.0%) | 0 (0%) |
| **Congestive heart failure** |  |  |  |
| No | 619 (89.7%) | 3819 (88.6%) | 29 (78.4%) |
| Yes | 71 (10.3%) | 491 (11.4%) | 8 (21.6%) |
| Missing | 0 (0%) | 2 (0.0%) | 0 (0%) |
| **Peripheral vascular disease** |  |  |  |
| No | 651 (94.3%) | 4058 (94.1%) | 32 (86.5%) |
| Yes | 39 (5.7%) | 252 (5.8%) | 5 (13.5%) |
| Missing | 0 (0%) | 2 (0.0%) | 0 (0%) |
| **Stroke** |  |  |  |
| No | 601 (87.1%) | 3780 (87.7%) | 32 (86.5%) |
| Yes | 89 (12.9%) | 530 (12.3%) | 5 (13.5%) |
| Missing | 0 (0%) | 2 (0.0%) | 0 (0%) |
| **Neurocognitive disorder** |  |  |  |
| No | 675 (97.8%) | 4179 (96.9%) | 36 (97.3%) |
| Yes | 15 (2.2%) | 131 (3.0%) | 1 (2.7%) |
| Missing | 0 (0%) | 2 (0.0%) | 0 (0%) |
| **COPD** |  |  |  |
| No | 591 (85.7%) | 3657 (84.8%) | 28 (75.7%) |
| Yes | 99 (14.3%) | 653 (15.1%) | 9 (24.3%) |
| Missing | 0 (0%) | 2 (0.0%) | 0 (0%) |
| **Connective tissue disease** |  |  |  |
| No | 649 (94.1%) | 4145 (96.1%) | 34 (91.9%) |
| Yes | 41 (5.9%) | 165 (3.8%) | 3 (8.1%) |
| Missing | 0 (0%) | 2 (0.0%) | 0 (0%) |
| **Peptic ulcers** |  |  |  |
| No | 659 (95.5%) | 4133 (95.8%) | 32 (86.5%) |
| Yes | 31 (4.5%) | 177 (4.1%) | 5 (13.5%) |
| Missing | 0 (0%) | 2 (0.0%) | 0 (0%) |
| **Liver disease** |  |  |  |
| No | 676 (98.0%) | 4240 (98.3%) | 34 (91.9%) |
| Severe | 1 (0.1%) | 12 (0.3%) | 0 (0%) |
| Yes | 13 (1.9%) | 58 (1.3%) | 3 (8.1%) |
| Missing | 0 (0%) | 2 (0.0%) | 0 (0%) |
| **Diabetes** |  |  |  |
| No or under control | 503 (72.9%) | 3232 (75.0%) | 26 (70.3%) |
| Yes but no complications | 181 (26.2%) | 1034 (24.0%) | 11 (29.7%) |
| Yes, damage to organs | 6 (0.9%) | 44 (1.0%) | 0 (0%) |
| Missing | 0 (0%) | 2 (0.0%) | 0 (0%) |
| **Hemiplegia** |  |  |  |
| No | 686 (99.4%) | 4280 (99.3%) | 37 (100%) |
| Yes | 4 (0.6%) | 30 (0.7%) | 0 (0%) |
| Missing | 0 (0%) | 2 (0.0%) | 0 (0%) |
| **Chronic renal insufficiency** |  |  |  |
| No | 648 (93.9%) | 4045 (93.8%) | 35 (94.6%) |
| Yes | 42 (6.1%) | 265 (6.1%) | 2 (5.4%) |
| Missing | 0 (0%) | 2 (0.0%) | 0 (0%) |
| **Solid tumor** |  |  |  |
| Local | 170 (24.6%) | 1001 (23.2%) | 7 (18.9%) |
| Metastasized | 15 (2.2%) | 120 (2.8%) | 2 (5.4%) |
| None | 505 (73.2%) | 3189 (74.0%) | 28 (75.7%) |
| Missing | 0 (0%) | 2 (0.0%) | 0 (0%) |
| **Leukemia** |  |  |  |
| No | 685 (99.3%) | 4269 (99.0%) | 37 (100%) |
| Yes | 5 (0.7%) | 41 (1.0%) | 0 (0%) |
| Missing | 0 (0%) | 2 (0.0%) | 0 (0%) |
| **Lymphoma** |  |  |  |
| No | 680 (98.6%) | 4246 (98.5%) | 37 (100%) |
| Yes | 10 (1.4%) | 64 (1.5%) | 0 (0%) |
| Missing | 0 (0%) | 2 (0.0%) | 0 (0%) |
| **AIDS** |  |  |  |
| Non | 690 (100%) | 4306 (99.9%) | 37 (100%) |
| Yes | 0 (0%) | 4 (0.1%) | 0 (0%) |
| Missing | 0 (0%) | 2 (0.0%) | 0 (0%) |
| **Race** |  |  |  |
| Caucasian | 689 (99.9%) | 4297 (99.7%) | 37 (100%) |
| Missing | 1 (0.1%) | 4 (0.1%) | 0 (0%) |
| Afro-Canadian | 0 (0%) | 4 (0.1%) | 0 (0%) |
| First Nations | 0 (0%) | 3 (0.1%) | 0 (0%) |
| Other | 0 (0%) | 3 (0.1%) | 0 (0%) |
| Hispanic | 0 (0%) | 1 (0.0%) | 0 (0%) |
| **First Language** |  |  |  |
| English | 2 (0.3%) | 26 (0.6%) | 0 (0%) |
| French | 688 (99.7%) | 4276 (99.2%) | 37 (100%) |
| Other | 0 (0%) | 7 (0.2%) | 0 (0%) |
| Spanish | 0 (0%) | 1 (0.0%) | 0 (0%) |
| Unknown or missing | 0 (0%) | 2 (0.0%) | 0 (0%) |
| **Household Income** |  |  |  |
| < 10 000$ | 16 (2.3%) | 95 (2.2%) | 1 (2.7%) |
| 10 000 - 19 999$ | 103 (14.9%) | 631 (14.6%) | 9 (24.3%) |
| 20 000 - 29 999$ | 120 (17.4%) | 793 (18.4%) | 8 (21.6%) |
| 30 000 - 39 999$ | 90 (13.0%) | 561 (13.0%) | 3 (8.1%) |
| 40 000 - 49 999$ | 62 (9.0%) | 319 (7.4%) | 1 (2.7%) |
| 50 000 - 59 999$ | 34 (4.9%) | 192 (4.5%) | 2 (5.4%) |
| 60 000 - 69 999$ | 16 (2.3%) | 120 (2.8%) | 1 (2.7%) |
| 70 000 - 79 999$ | 9 (1.3%) | 80 (1.9%) | 0 (0%) |
| 80 000 - 89 999$ | 7 (1.0%) | 50 (1.2%) | 0 (0%) |
| 90 000 - 99 999$ | 8 (1.2%) | 27 (0.6%) | 0 (0%) |
| > 100 000$ | 15 (2.2%) | 113 (2.6%) | 0 (0%) |
| Missing or unknown | 25 (3.6%) | 102 (2.4%) | 1 (2.7%) |
| Did not wish to respond | 185 (26.8%) | 1229 (28.5%) | 11 (29.7%) |
| **Highest Level of Education** |  |  |  |
| Baccalaureate | 51 (7.4%) | 362 (8.4%) | 1 (2.7%) |
| College | 75 (10.9%) | 444 (10.3%) | 5 (13.5%) |
| Graduate school | 20 (2.9%) | 137 (3.2%) | 3 (8.1%) |
| Primary school | 313 (45.4%) | 1876 (43.5%) | 14 (37.8%) |
| Secondary school | 178 (25.8%) | 1085 (25.2%) | 10 (27.0%) |
| Professional diploma (DEP, ASP) | 52 (7.5%) | 388 (9.0%) | 4 (10.8%) |
| Did not wish to respond | 0 (0%) | 1 (0.0%) | 0 (0%) |
| Missing | 1 (0.1%) | 19 (0.4%) | 0 (0%) |
| **Residence** |  |  |  |
| Home, with others | 394 (57.1%) | 2619 (60.7%) | 22 (59.5%) |
| Home, alone | 198 (28.7%) | 1091 (25.3%) | 6 (16.2%) |
| Low-income housing | 9 (1.3%) | 42 (1.0%) | 0 (0%) |
| Unknown or missing | 3 (0.4%) | 6 (0.1%) | 0 (0%) |
| Care home with 24hr nurse | 54 (7.8%) | 365 (8.5%) | 5 (13.5%) |
| Care home | 27 (3.9%) | 182 (4.2%) | 4 (10.8%) |
| Family type resources | 4 (0.6%) | 2 (0.0%) | 0 (0%) |
| Other | 1 (0.1%) | 5 (0.1%) | 0 (0%) |
| **Social Support (# of persons)** |  |  |  |
| Mean (SD) | 3.79 (3.74) | 4.02 (4.01) | 2.41 (1.67) |
| Median [Min, Max] | 3.00 [0, 60.0] | 3.00 [0, 150] | 2.00 [0, 8.00] |
| Missing | 1 (0.1%) | 1 (0.0%) | 0 (0.0%) |
| **Have family MD** |  |  |  |
| No | 58 (8.4%) | 324 (7.5%) | 4 (10.8%) |
| Yes | 632 (91.6%) | 3986 (92.4%) | 33 (89.2%) |
| Missing | 0 (0%) | 2 (0.0%) | 0 (0%) |
| **Can get appointment with family MD** |  |  |  |
| Don’t know | 24 (3.5%) | 124 (2.9%) | 3 (8.1%) |
| No | 258 (37.4%) | 1459 (33.8%) | 11 (29.7%) |
| Yes | 357 (51.7%) | 2440 (56.6%) | 20 (54.1%) |
| Missing | 51 (7.4%) | 289 (6.7%) | 3 (8.1%) |
| **Access to transport** |  |  |  |
| No | 41 (5.9%) | 289 (6.7%) | 2 (5.4%) |
| Yes | 649 (94.1%) | 4009 (93.0%) | 35 (94.6%) |
| Don’t know | 0 (0%) | 12 (0.3%) | 0 (0%) |
| Missing | 0 (0%) | 2 (0.0%) | 0 (0%) |
| **Have a caregiver** |  |  |  |
| No | 495 (71.7%) | 3105 (72.0%) | 28 (75.7%) |
| Yes | 195 (28.3%) | 1207 (28.0%) | 9 (24.3%) |

**HDL: Hôtel-Dieu de Lévis, HDM : Hôpital de Montmagny; HSG : Hôpital de Saint-Georges; HTM : Hôpital de Thetford-Mines**

Appendix C

**Themes, selected patient comments, their translations, and definitions of each theme.**

**(Note: These quotes are the written notes taken by the research professionals doing the phone interviews and the third-person point of view is used when describing what the patient had said. The first-person point of view is used when the research professional is quoting the patient’s own words.)**

| **Theme** | **Original comment** | **Translated Comment** | **Definition** |
| --- | --- | --- | --- |
| Sense of security at the ED | 3_214 : Les médecins lui ont dit : *“*Vous avez pas d’affaire à venir ici vous avez rien*”*, alors qu’il se sentait étouffé en se couchant. … Considère dangereux de se renvoyer à cette urgence.    3_1986 : Bien entourée par le personnel de l’urgence ; sentiment de sécurité…. | *3_214: The doctors told him, “You have no business coming here...you have nothing [wrong with you]”, even though was feeling as if he was suffocating as he went to bed. … [He] considers it dangerous to return to this emergency department.*    *3_1986: Well surrounded by [in good hands with] emergency personnel, I felt safe….* | Ability to prevent risks and preserve the health and well-being of the patients during care. |
| Explanations | 1_1368 : …beaucoup d’explication pour le retour à la maison.;    1_1612 : …pas d’explication sur quoi faire avec la fracture et la commotion… ;    2_796 : …Mais, les explications faut aller les chercher parce que les médecins sont trop pressés. | *1_1368: … lots of explanations for the return home.*    *1_1612: … no explanation of what to do with the fracture and the concussion…*    *2_796: … But the explanations, [you] must go and get them [yourself] because the doctors are in too much of a hurry [to give the patient explanations without having to ask].* | Have received all relevant information delivered in an appropriate language to understand the interventions, results, and follow-up care in the emergency department. |
| Professionalism | 1_1928 : Pas aimé l’attitude du médecin, reçue… comme un chien dans un jeu de quilles… A été négligée. | *1_1928: Disliked [the] physician’s attitude, received… like a dog in a bowling game… [was poorly greeted] Neglected.* | Ability of ED staff to act professionally towards the patient. Reflects having respect, active listening skills, and compassion for the presenting patient. |
| Accompaniment to the ED | 1_1733: …sa fille avec lui pour les explications du retour…    1_1024 : Mon gendre ambulancier était avec moi, tout à bien été. | *1_1733: … his daughter with him to explain the return [home]…*    *1_1024: My son-in-law was with me, all was well.* | Have a familiar and trusted person accompany the patient during the stay at the ED and during the transition of care. |
| Communications in care | 1_4307 : … Mauvaise communication pour les examens complémentaires…    2_2281 : …Elle est partie en ambulance de l’hôpital XX pour aller à l’hôpital YY pour des examens d’ORL. Son médecin de XX lui avait dit qu’elle reviendrait à XX en ambulance pour qu’elle puisse reprendre son véhicule, mais une fois à YY, on lui a dit qu’elle devrait s’organiser toute seule pour son transport de retour. Elle a été prise au dépourvu, sans moyen de transport de retour. | *1_4307: … Miscommunication for additional exams…*    *2_2281: … She went by ambulance from hospital XX to hospital YY for ENT exams. Her doctor at XX had told her that she would return to hospital XX by ambulance so that she could take her vehicle back, but once at YY, she was told that she would have to plan herself for her return transportation. She was caught unaware, with no means of return transportation.* | Transfer of the patient’s medical information (family history, test results, level of care, etc.) to all relevant stakeholders within an acceptable time frame. |
| Clinical intervention | 2_1757: … mais mauvaise manipulation lors de transferts…    1_3451 : Problème avec piqûres dans son bras droit et gauche (saignement pendant 15 jours). Il considère que l’équipe de médecin n’était pas qualifiée pour son cas… Ils ne comprenaient pas ce qu’il avait. Il était déçu de sa visite à l’urgence. | *2_1757: … but bad handling when transferring…*    *1_3451: Problem with venous punctures in right and left arm (bleeding for 15 days). He felt that the medical team was not qualified for his case.... They didn’t understand what he had. He was disappointed with his visit to the emergency department.* | Specific medical care and techniques performed in the hospital. |
| Sense of security at home | 1_3449: Les infirmières viennent à tous les jours. Elle se sent en sécurité.    1_663 : J’ai une infirmière au CLSC qui vient mais si non tout va bien, je suis encore autonome et j’ai ma femme. | *1_3449: Nurses visit every day. She feels safe.*    *1_663: I have a nurse at the CLSC who visits but other than that, everything is going well, I am still independent, and I have my wife.* | Ability to feel sheltered from danger, confident, safe, and at peace in one’s living environment. |
| Home care | 3_2004 : … Sa pharmacie livre les médicaments et elle apprécie beaucoup ce service.    1_7 : …Le CLSC ouvrait seulement le lundi suivant, donc sans la marchette de sa mère, il n’aurait pas eu accès à aucun équipement pour l’aider à se déplacer dans la maison durant la fin de semaine. Il mentionne que les soins et le matériel offert par l’ergothérapeute l’a toutefois beaucoup aidé. | *3_2004: … Her pharmacy delivers medication, and she really appreciates this service.*    *1_7: … The CLSC [local community services centre] opened only the following Monday, so without his mother’s walker, he would not have had access to any equipment to help him move around the house on the weekend. He mentioned that the care and equipment offered [to him] by the occupational therapist helped him a lot.* | Intervention or service performed outside of a hospital center (home, community clinic, pharmacy, etc.) which requires specific training or accreditation. For instance, home visits by a nurse, physician, social worker were considered part of home care. So too were appointments with a nutritionist, optometrist, occupational therapist, and consultations with a community pharmacist. |
| Isolation / Socialization | 2_643 : … mais par après ils l’ont condamnée dans son appartement car elle avait l’influenza. Pognée avec ses vidanges, ils ne venaient pas les chercher. Pouvait pas sortir sans que la chambre soit désinfectée… même pas ouvrir la porte….    1_7 : …Monsieur mentionne qu’il réside seul et que s’il n’avait pas gardé une marchette de sa mère décédée, il aurait dû ramper sur le sol de la maison pour se déplacer. Heureusement, une amie a offert son aide. Elle est allée chercher sa prescription de morphine à la pharmacie, sans quoi il aurait été seul et sans médication… | *2_643: … but then they condemned her to her apartment because she had influenza. Stuck with her garbage, [no one came to take out her garbage]. [She] couldn’t leave without the room being disinfected…not even [to open] the door….*    *1_7: … [He] mentions that he lives alone and that if he had not kept a walker from his [deceased] mother, he would have had to crawl around the floor of the house to move. Fortunately, a friend offered to help. She went to the pharmacy to get his morphine prescription, otherwise he would have been alone and without medication...* | Ability to have support from a social network or another person. |
| Domestic help | 3_801 : … Les aides au ménage et bouffe c’est 1 an et plus d’attente peu importe ta condition physique. Elle a tout fait pour avoir de l’aide mais l’attente est vraiment longue. Il devrait y avoir des priorités….    1_3327 : Elle me dit qu’elle aimerait avoir une aide supplémentaire à la maison pour les tâches. | *3_801: … Support for cleaning and food preparation at home [takes one year or longer on a waiting list] regardless of your physical condition. She has done everything she can to get help, but the wait is really long. There should be priorities...*    *1_3327: She tells me that she would like additional help at home with tasks.* | Someone who can offer help with daily chores and household tasks. |
| Privacy | 2_586 : … Manque d’intimité lors des consultations…    1_1361 : Manque de personnel, difficile à avoir de l’aide si besoin urgent.    1_2841 : Une seule chose que je n'ai pas aimé : la petite chaise pour les toilettes… | *2_586: … Lack of privacy during consultations…*    *1_1361: Lack of staff, difficult to get help [going to the toilet] if you urgently need to go.*    *1_2841: One thing I didn’t like: the little chair for the toilet…* | Ability to be comfortable in private moments and interacting with the person(s) administering care by considering the surrounding conditions. |
| Sleeping conditions | 1_7 : … Il mentionne que les infirmières discutaient fort en pleine nuit et il n’a pas été capable de dormir.    1_197 : Monsieur mentionne que lors de son hospitalisation, il a été déménagé de sa chambre durant la nuit. Mentionne ne pas avoir été averti et il a passé la nuit dans le corridor… | *1_7: … He mentioned that the nurses were chatting loudly in the middle of the night, and he was unable to sleep.*    *1_197: [He] mentioned that during his hospitalization, he was moved out of his room overnight. Mentions not being notified and ended up spending the night in the hallway…* | Ability to ensure adequate conditions for rest and to respect patients’ day and night cycles. |
| Bedding, clothing, and furniture | 2_2281 : …Elle mentionne également que les lits à l’urgence XX étaient très inconfortables, ce qui l’a beaucoup fait souffrir puisqu’elle y est restée longtemps.    1_1143 : Ils m’ont laissé sortir en jaquette 2 heures sur le banc dehors. | *2_2281: … She also mentioned that the beds in the XX emergency room were very uncomfortable, which made her suffer a lot because she was bedridden for a long time.*    *1_1143: They let me out in a hospital gown for 2 hours on the bench outside.* | Have furniture, bedding, bedclothes, and accommodations adapted to the seasons and to the patients who use them. |
| Hygiene and sanitation | 1_1928 : Il est resté longtemps dans ses selles sans se faire changer.    1_2536 : … doute sur la propreté au niveau de l’entretien de ses draps…    4_820 : … (patient a beaucoup saigné après une prise de sang) (infirmière) est allé le voir pas ganté, elle a pris ses doigts directs. Il a dit “t’es pas sensée mettre des gants.*”* Elle a dit “pas grave, vous avez pas le SIDA vous.*”* … Dans la nuit, il a eu soif, l’infirmière lui a amené un verre d’eau en le tenant où le goulot au lieu de par la base… | *1_1928: Remained [sitting in his stool soiled clothes] for a long time without being changed.*    *1_2536: … [had] doubt[s] about cleanliness in the care of [his] bedsheets...*    *4_820: … (patient bled a lot after a blood test) [and the] (nurse) went to see him without gloves. He asked, “you’re not supposed to wear gloves?” She said “no, you don’t have AIDS…”. During the night, he got thirsty, and the nurse brought him a glass of water holding the glass by the neck instead of by the base…* | Have a healthy environment and the hospital staff implement appropriate prophylactic behaviors to promote recovery. |
| Security in accommodation | 3_1564 : … (patiente sans globule blancs) Avait un peu peur puisqu’elle restait dans un couloir avec un rideau, n’était pas certaine que l’infirmière changeait ses gants entre ses patients. Aurait aimé mieux avoir une chambre à part et que l’infirmière change ses gants devant elle avant de l’examiner.    3_2081 : En pleine pandémie, ce n’est pas le temps de se faire appeler pour des rendez-vous !! Je me fais appeler pour toutes sortes d’affaires à aller passer; … ce n’est pas le temps de faire ça, on doit rester chez nous pis ne pas sortir !!! C’est dangereux à l’hôpital, à chaque fois que je suis allé à l’urgence, les infirmières de la zone verte ne sont même pas à 30 pieds de ceux de la zone rouge, venez pas me faire croire que c’est pas dangereux. | *3_1564: … (patient without white blood cells) Was a little scared as she stayed in a hallway with a curtain, was not sure the nurse changed her gloves between patients. Would have preferred to have a separate room and [witness] the nurse change her gloves in front of [the patient] before examining [the patient].*  *3_2081: In the middle of a pandemic, now is not the time to call for appointments!!! I [get] calls for all kinds of things to come in for…this is not the time to do that, we must stay at home and not go out!!! It’s dangerous in the hospital, every time I’ve been to the emergency room, the nurses in the green zone aren’t even 30 feet away from the nurses in the red zone, don’t [try to convince me that] it’s not dangerous.* | Ability to feel safe from danger during accommodation. |
| Food | 1_334: … Au moment du repas, il a été oublié et la nourriture laisse à désirer.    1_3653 : Très long et très pénible sans manger et boire durant 24h… | *1_334: … At mealtime, I was forgotten, and the food left [much] to be desired.*    *1_3653: Very long and very painful without eating and drinking for 24 hours…* | Ability to provide good quality, appetizing food and hydration adapted to patient needs. |
| Sense of security during discharge | 1_1349: Aucune réponse au final, rien trouvé, mais je me sentais en sécurité de retourner chez moi.    2_971 : Attente : arrivé à 11h à 19h, ils leur ont annoncé que l’urgence fermait pour 4h. Retour chez lui puis retourne à l’urgence à 23h15. L’urgence a dû fermer parce que le médecin devait accompagner un patient jusqu’à Québec en ambulance. | *1_1349: No answer in the end, [they did not find anything wrong], but I felt safe to return home.*    *2_971: Waiting: arrived at 11am to 7pm, they announced that the emergency was closing for 4 hours. Return[ed] home and return[ed] to the emergency department at 11:15pm. The emergency department had to close because the doctor had to accompany a patient to Quebec City by ambulance.* | Feeling safe from danger while returning to their home living environment. |
| Transport for medical care | 1_7: … lors de sa sortie, il avait besoin d’un transport assisté. ... Il mentionne avoir demandé un transport assisté mais que le personnel n’arrivait pas à lui en trouver un. Finalement, son gendre est venu l’aider et alors qu’il était sur le point de quitter, la secrétaire du CLSC lui a mentionné qu’un transport avait été demandé. Elle lui a mentionné que même s’il ne le prenait pas, il allait devoir payer les frais associés. Monsieur a pris le transport... (autre visite) Son transport devait arriver à 17h30 et il est finalement arrivé à 19h30.    2_2274 : … Elle n’avait pas non plus de transport pour retourner chez elle, car elle est arrivée en ambulance et personne ne voulait venir la chercher puisqu’elle était positive à la COVID-19. | *1_7: … on his way out, he needed assisted transportation. ... He mentioned that he requested assisted transportation but that the staff could not find one [for him]. Finally, his son-in-law came to help him and while he was about to leave, the secretary of the CLSC told him that transportation had been requested. She told him that even if he did not take [the assisted transportation], he would have to pay the associated costs. He took the transportation.... (another visit) His transport was supposed to arrive at 5:30 PM and it only arrived at 7:30 PM.*    *2_2274: … She also did not have transportation to go home because she arrived in an ambulance, and no one wanted to pick her up because she [tested] positive for COVID-19.* | Ability to have transportation or a vehicle for attending appointments related to their medical condition. |
| Departure | 4_664 : … médecin … n’a pas regardé à la bonne place. Il l’a renvoyé chez lui et il a été obligé de revenir à l’hôpital le lendemain en ambulance…    2_252 : renvoyé chez lui, a dû retourner au bout de 2 jours, c’est arrivé 3 fois ce cycle là…    1_1742 : … ils l’ont mis dehors. Ils ne les gardent pas plus longtemps qu'il le faut. | *4_664: … doctor … did not look in the right place. [The doctor] sent him home and [the patient] had to return to hospital the next day by ambulance…*    *2_252: sent home, had to return after 2 days, [this cycle] has happened 3 times …*    *1_1742: … They kicked him out. They don’t keep them [let patients stay] any longer than they need to.* | Circumstance surrounding the conclusion of hospital care and the return to the patient’s usual living environment. |

**Appendix D**

**Standards for Reporting Qualitative Research (SRQR)***

http://www.equator-network.org/reporting-guidelines/srqr/

**Page or line #s**

| **Title** - Concise description of the nature and topic of the study. Identifying the study as qualitative or indicating the approach (e.g., interview, focus group) is recommended | Page 1 |
| --- | --- |
| **Abstract** - Summary of key elements of the study using the abstract format of the intended publication; typically includes background, purpose, methods, results, and conclusions | Page 1 |

**Introduction**

| **Problem formulation** - Description and significance of the problem/phenomenon studied; review of the relevant theory and empirical work; problem statement | Page 3 |
| --- | --- |
| **Purpose or research question** - Purpose of the study and specific objectives or questions | Bottom of Page 3 and Start of Page 4 |

**Methods**

| **Qualitative approach and research paradigm** - Qualitative approach (e.g., ethnography, grounded theory, case study, phenomenology, narrative research) and guiding theory if appropriate; identifying the research paradigm (e.g., postpositivist, constructivist/ interpretivist) is also recommended; rationale** | Page 4, Page 6, |
| --- | --- |
| **Research characteristics and reflexivity** - Researchers’ characteristics that may influence the research, including personal attributes, qualification/experience, relationship with participants, assumptions, and/or presuppositions; potential or actual interaction between researchers’ characteristics and the research questions, approach, methods, results, and/or transferability | Page 6 |
| **Context** - Setting/site and salient contextual factors; rationale** | Selection, Page 4 |
| **Sampling strategy** - How and why research participants, documents, or events were selected; criteria for deciding when no further sampling was necessary (e.g., sampling saturation); rationale** | Sampling, Page 8  Saturation, Page 10 |
| **Ethical issues pertaining to human subjects** - Documentation of approval by an appropriate ethics review board and participant consent, or explanation for lack thereof; other confidentiality and data security issues | Study design and context, Page 4 |
| **Data collection methods** - Types of data collected; details of data collection procedures including (as appropriate) start and stop dates of data collection and analysis, iterative process, triangulation of sources/methods, and modification of procedures in response to evolving study findings; rationale** | Page 5,  Data collection,  Pages 6 & 7 |
| **Data collection instruments and technologies** - Description of instruments (e.g., interview guides, questionnaires) and devices (e.g., audio recorders) used for data collection, if/how the instruments(s) changed over the course of the study | Constructing the coding scheme, Pages 8-9 |
| **Units of study** - Number and relevant characteristics of participants, documents, or events included in the study; level of participation (could be reported in results) | Page 11,  Recruitment flowchart on Page 13, Table 1 on Pages 14-17 |
| **Data processing** - Methods for processing data prior to and during analysis, including transcription, data entry, data management and security, verification of data integrity, data coding, and anonymization/de-identification of excerpts | Pages 6-8, 10 |
| **Data analysis** - Process by which inferences, themes, etc., were identified and developed, including the researchers involved in data analysis; usually references a specific paradigm or approach; rationale** | Pages 8-10 |
| Techniques to enhance trustworthiness - Techniques to enhance trustworthiness and credibility of data analysis (e.g., member checking, audit trail, triangulation); rationale** | Reliability described on Page 9. |

**Results/findings**

| **Synthesis and interpretation** - main findings (e.g., interpretations, inferences, and themes); might include development of a theory or model. Or integration with prior research or theory | Pages 11-24 |
| --- | --- |
| **Links to empirical data** - Evidence (e.g., quotes, field notes, text excerpts, photographs) to substantial analytic findings | Appendix C |

**Discussion**

| **Integration with prior work, implications, transferability, and contribution(s) to field** - short summary of main findings; explanation of how findings and conclusions connect to, support, elaborate on, or challenge conclusions of earlier scholarship; discussion of scope of application/generalizability; identification of unique contribution(s) to scholarship in a discipline or field | Pages 26-29 |
| --- | --- |
| **Limitations** - Trustworthiness and limitations of findings | Pages 29-31 |

**Other**

| **Conflicts of interest** - Potential sources of influence or perceived influence on study conduct and conclusions; how these were managed | Page 33 |
| --- | --- |
| **Funding** - Sources of funding and other support; role of funders in data collection, interpretation, and reporting | Page 33 |
